# Investigation of Various Active Layers for Their Performance on Organic Solar Cells

**DOI:** 10.3390/ma9080667

**Published:** 2016-08-09

**Authors:** Pao-Hsun Huang, Yeong-Her Wang, Jhong-Ciao Ke, Chien-Jung Huang

**Affiliations:** 1 Institute of Microelectronics, Department of Electrical Engineering, National Cheng Kung University, Tainan 70101, Taiwan; Q18031035@mail.ncku.edu.tw (P.-H.H.); YHW@eembox.ncku.edu.tw (Y.-H.W.); m0984307@mail.nuk.edu.tw (J.-C.K.); 2 Department of Applied Physics, National University of Kaohsiung, Nanzih, Kaohsiung 81148, Taiwan; chien@nuk.edu.tw

**Keywords:** small molecule organic solar cells, open-circuit voltages, thermal vacuum evaporation deposition

## Abstract

The theoretical mechanism of open-circuit voltages (V_OC_) in OSCs based on various small molecule organic materials is studied. The structure under investigation is simple planar heterojunction (PHJ) by thermal vacuum evaporation deposition. The various wide band gaps of small molecule organic materials are used to enhance the power conversion efficiency (PCE). The donor materials used in the device include: Alpha-sexithiophene (α-6T), Copper(II) phthalocyanine (CuPc), boron subnaphthalocyanine chloride (SubNc) and boron Subphthalocyanine chloride (SubPc). It is combined with fullerene or SubPc acceptor material to obtain a comprehensive understanding of the charge transport behavior. It is found that the V_OC_ of the device is largely limited by charge transport. This was associated with the space charge effects and hole accumulation. These results are attributed to the improvement of surface roughness and work function after molybdenum trioxide (MoO_3_) is inserted as an anode buffer layer.

## 1. Introduction

A big milestone is being reached by organic solar cells (OSCs) because they have potential and are a conceivable alternative to traditional inorganic solar cells due to their ease of processing and compatibility with flexible substrates [1,2]. The OSCs have attracted significant industrial and academic interest and research as a promising source of renewable global energy today. At present, one of the most important issues is how to achieve the high power conversion efficiency (PCE) of solar cells through sample, solution and large area manufacturing methods. Following the introduction of the donor/acceptor (DA) interface by Tang, both the bulk heterojunction and tandem structure have been the most explored device architectures to enhance PCE [3,4,5]. The development of improved novel materials is another essential factor to further increase efficiency. The PCE of OSCs is improved continually by designing novel materials and device architectures for matching energy level in the active layer. One of the major challenges to obtaining high efficiency in OSCs is the low open-circuit voltage (V_OC_), which is restricted by the energy offset between the highest occupied molecule orbital (HOMO) of the electron donor and the lower unoccupied molecule orbital (LUMO) of the electron acceptor [6]. In 2010, Tang and co-workers published a study relating to Schottky barrier photovoltaic cells based on MoO_X_/C_60_ and realized a high V_OC_ of 1.23 V with a simple bilayer structure of ITO/MoO_X_/C_60_/Bathophenanthroline (BPhen)/LiF/Al [7]. However, the PCE of the device is only about 0.5%. Finally, it was found that the Schottky barrier can provide DA heterojunction to dissociate the exciton to obtain more photocurrent and high Voc. On the other hand, fullerene molecules and their derivatives have become the primary acceptor materials in organic photovoltaic cells because of their better electron-accepting ability and high electron mobility [8,9]. The small absorption is due to overlap with the solar spectrum by using fullerene as an acceptor, which might be the reason that the photocurrent is limited. In addition, the small energy band gap also limits the V_OC_ in the device [10].

**Figure 1:**
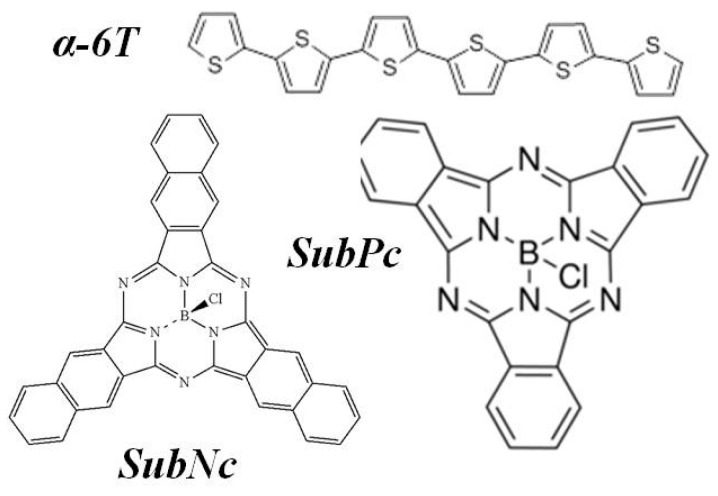
The molecule chemical structure of the used materials and schematic representation of the device structure.

In this study, we accomplished the bilayer device and measure the performance of Schottky barrier cell by replacing fullerene with SubPc or SubNc as the active layer through thermal vacuum evaporation deposition. We also examine a simple three-layer architecture comprising various organic materials as donor and acceptor, where an energy-relay cascade enables an efficient two-step exciton dissociation process. Figure 1 shows the chemical structure of the materials and a schematic diagram of the device structure. Finally, we obtained a short-circuit current (J_SC_) of 9.88 mA/cm^2^, a V_OC_ of 0.94 V, and a PCE of 4.35% with the three-layer device structure: ITO-Glass/MoO_3_/α-6T/SubNc/SubPc/BCP/Ag.

## 2. Results

**Figure 2:**
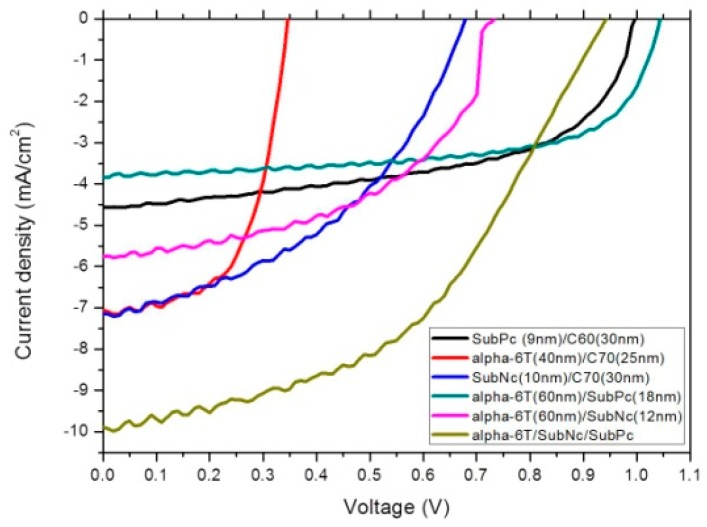
The current density-voltage (J-V) characteristic of these devices with various donor and acceptor materials.

**Figure 3:**
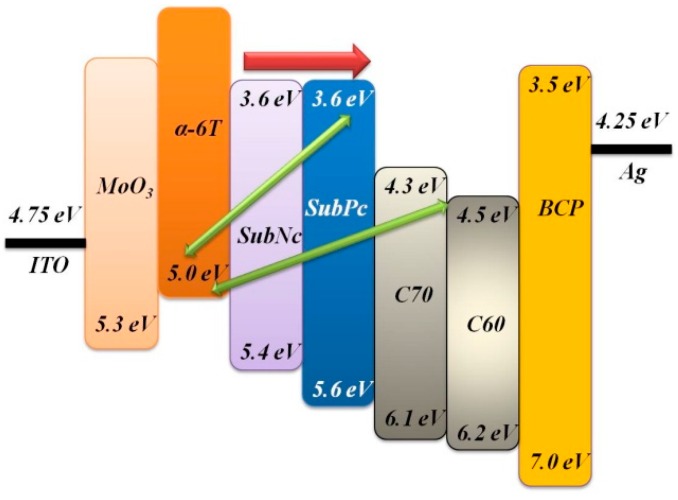
The energy levels diagram of used materials illustrating the charge transfer mechanism in this study.

**Figure 4:**
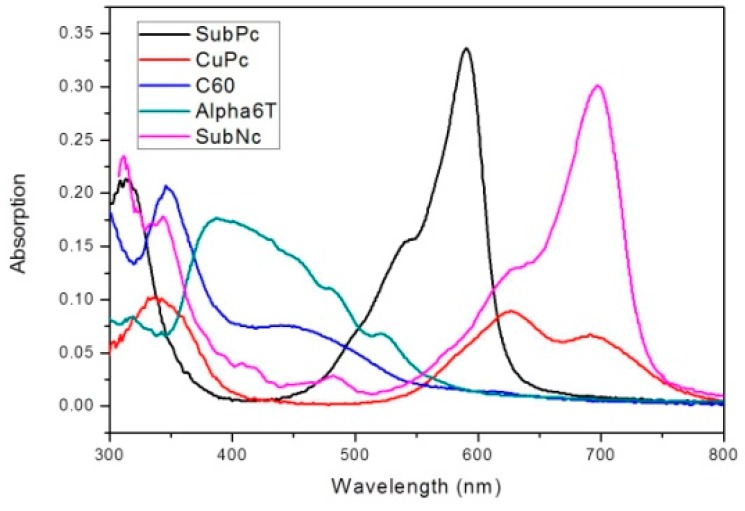
The thin-film optical absorption spectrum of used materials from 300 nm to 800 nm to effectively harvest the solar light.

**Figure 5:**
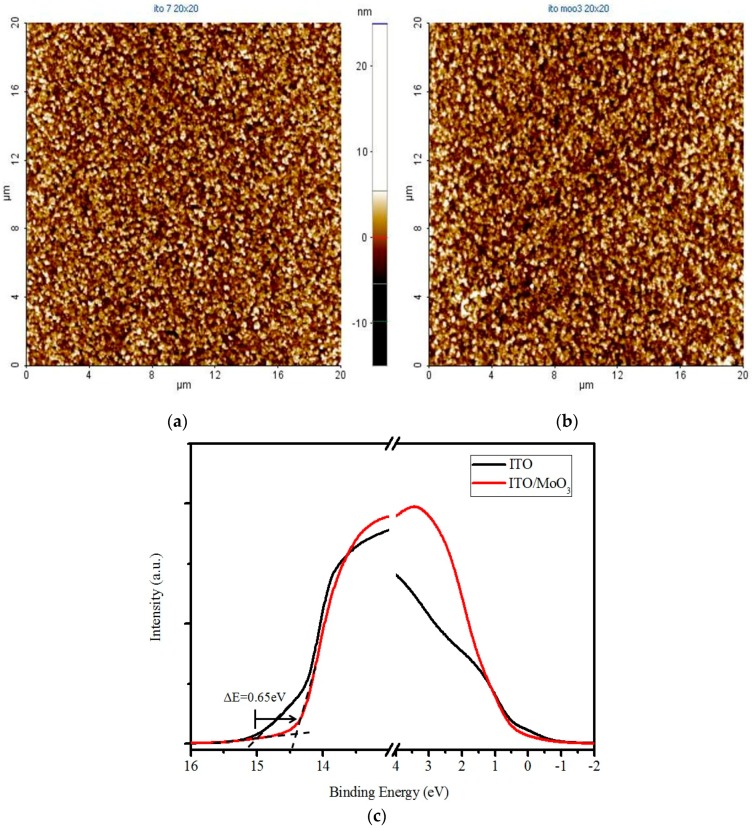
The result of surface roughness: (**a**) The device with the anode buffer layer; (**b**) The device without the anode buffer layer; (**c**) The ultraviolet photoelectron spectroscopy (UPS) spectrum of indium tin oxide (ITO) and ITO/MoO_3_.

Bilayer devices were fabricated on indium tin oxide-coated glass substrates with sheet resistance of 7 Ω/sq. For the following layer structure: Molybdenum trioxide (MoO_3_)/Donor material/Fullerene (C_60_,C_70_)/Bathocuproine (BCP)/Ag, where the donor material is alpha-sexithiophene (α-6T), Copper(II) phthalocyanine (CuPc), boron subnaphthalocyanine chloride (SubNc) and boron Subphthalocyanine chloride (SubPc), Figure 2 shows the current density-voltage (J-V) characteristic of these device with various donor and acceptor materials. The energy levels of materials used are shown in the Figure 3. It explains the theoretical mechanism of V_OC_ between donor and acceptor. Figure 4 shows the thin-film optical absorption spectrum of materials used from 300 nm to 800 nm. Figure 5 revealed the surface roughness with and without the anode buffer layer. The photovoltaic performance of bilayer OSCs (Donor/C_60_) are listed in the Table 1. Both Table 2 and Table 3 show the photovoltaic result based on α-6T donor material by using different acceptor material. Finally, the performance of three-layer cascade device based on following structure: ITO/MoO_3_/α-6T/SubNc/ SubPc/BCP/Ag is shown in Table 4.

## 3. Discussion

The thickness of the active layer was optimized to obtain an excellent PCE for each device. The thickness of the BCP cathode buffer layer and MoO_3_ anode buffer layer is fixed at 10 nm, respectively. In general, to get the better efficiency of OSCs, inserting the cathode buffer layer between the C_60_ and metal cathode is necessary. The electron transport across the cathode buffer layer mainly occurs via states induced in the cathode buffer layer during the cathode deposition. Most of the small molecular OSCs employ BCP or bathophenanthroline (Bphen) as a cathode buffer layer. In Figure 5a,b, the AFM measurements show the decreased roughness of ITO from 3.555 ± 0.045 to 2.735 ± 0.085 nm by covering the evaporated MoO_3_ layer. The AFM images simultaneously showed the difference from ITO and ITO/MoO_3_ in 2-dimensional (2D) top view to further understand the nanostructure. It is found that the roughness is improved by evaporating an MoO_3_ layer of 10 nm, although the ITO surface is not smooth because clear and dispersive white spots can be seen in Figure 5a. In other words, the dissociated exciton would fluently transport to an electrode when the MoO_3_ layer is deposited on the ITO surface, corresponding to the white spots gathering to become an island in the Figure 5b. On the other hand, it showed that the function of MoO_3_ not only acted as an exciton-blocking layer but also modified the work function of ITO [11,12,13]. In Figure 5c, the UPS spectrum showed the difference from ITO and ITO/MoO_3_. The work function of ITO was improved from 4.75 to 5.4 eV by using the MoO_3_ as a buffer layer. In the binding energy spectrum, the MoO_3_ deposited on ITO caused a 0.65 eV energy shift of secondary electron cutoff for lower binding energy, implying that the work function increased 0.65 eV by depositing an MoO_3_ layer on the ITO substrate. In the energy level diagram, the charge transport can be improved by depositing an MoO_3_ layer on the ITO substrate. Furthermore, light harvesting is another reason to obtain better performance, which is shown in Figure 3 and Figure 4. The LUMO energy level of C_60_ and C_70_ is −4.5 eV and −4.3 eV, and there was a closed HOMO energy level of −6.1 eV and −6.2 eV, respectively. The value of energy level difference between the HOMO energy level of the donor and LUMO energy level of the acceptor could be predicted in Figure 2. The structure of the CuPc/C_60_, SubPc/C_60_, α-6T/C_60_ and α-6T/C_70_ device is 0.7 eV, 1.1 eV, 0.7 eV and 0.5 eV, respectively. This reflects the V_OC_ of the device from Table 1 to Table 3. The V_OC_ of the SubPc/C_60_ device (0.5 V) is higher than that of the other devices, and it has a better efficiency of 1.3%, shown in Table 1. In out theorem, the maximal value of the V_OC_ is associated with the energy level difference between the highest occupied molecular orbital of the donor (HOMO_D_) and the lowest unoccupied molecular orbital of the acceptor (LUMO_A_), but in fact the V_OC_ of a device is determined according to the difference in the work function of the electrodes [14,15,16]. The HOMO and LUMO are not discrete energy levels, but are instead defined from a density of states with a disorder induced energy distribution, therefore suggesting that recombination losses mainly take place at the donor/acceptor interface. In other words, the work function improvement of ITO/MoO_3_ also enhances the built-in electric field of the device to increase the carrier collection. More detail about the investigation of V_OC_ is related to the following equation:

(1) $$V_{\text{OC}}=\frac{1}{e}\left({\text{LUMO}}_{A}-{\text{HOMO}}_{D}-\Delta \right)-\frac{\text{KT}}{e}\ln\left(\frac{n_{e}n_{h}}{N_{c}^{2}}\right)$$

where the n_e_ and n_h_ are the electron and hole densities in the C_60_ and donor at open circuit; K is Boltzmann constant; Δ is the energy shift; T is the Kelvin scale and N_c_ is the density of conduction states at the band edge of C_60_ and donor (assume equal here). The commonly accepted value, V_OC_ ≒ (LUMO_A_ − HOMO_D_), is obtained from the above equation only at T = 0 K. The validity of the first term in equation has been verified for a number of 0.3 V of previously unknown origin [17,18,19]. At finite T, because of the fundamental statistics of Fermions, the quasi-Fermi levels move away from LUMO_A_ and HOMO_D_, respectively, and into the gap above the polymer HOMO energy level and LUMO energy level. The resulting reduction in V_OC_ is given by the second term in the equation and is the origin of the “missing 0.3 V” [17]. The efficiency of α-6T/C_70_ device is 1.44% which is better than that of other devices. This is attributed to the α-6T having excellent mobility and a broad absorption spectrum (As shown in the Figure 4). The absorption spectrum of BCP is below 350 nm, which is consistent with the BCP energy gap (3.5 eV). Furthermore, the approximate distribution of the absorption spectrum of another material is as follows: 300–400 nm and 600–700 nm for CuPc, 500–600 nm for SubPc, 350–550 nm for α-6T and 300–400 nm for C_60_. The small absorption overlap is the reason why the bilayer device based on the fullerene acceptor limited the efficiency and short-circuit density (J_SC_) performance.

Another method to increase the photocurrent in OSCs is incorporation of additional light-absorbing materials. Compared with the device based on fullerene in Table 1 and Table 2, both V_OC_ and J_SC_ are significantly improved for devices with SubPc and SubNc as an acceptor in Table 3. The device based on C_60_ acceptor in Table 1 achieved its best performance by using SubPc material with J_SC_ of 4.37 mA/cm^2^, V_OC_ of 0.55 V and PCE of 1.3%. The α-6T/C_70_ device in Table 2, however, has its best performance with J_SC_ of 7.05 mA/cm^2^, V_OC_ of 0.35 V and PCE of 1.44%. The SubNc/C_70_ device has its best performance with J_SC_ of 7.15 mA/cm^2^, V_OC_ of 0.68 V and PCE of 2.15%. The improvement of PCE from 1.44% to 2.15% is attributed to the increase of V_OC_ from 0.5 eV for α-6T/C_70_ device to 0.9 eV for SubNc/C_70_ device. The non-fullerene concept has been established to further enhance the PCE of OSCs. We found that devices based on the α-6T as donor have excellent performance compared to fullerene-based cells by using different acceptor materials SubPc and SubNc. Both SubPc and SubNc have a LUMO energy level of −3.6 eV. It is obvious in Table 3 that the α-6T/SubPc device has better performance than the fullerene-based cells with J_SC_ of 3.83 mA/cm^2^, V_OC_ of 1.04 V and PCE of 2.56%. Both V_OC_ and J_SC_ are significantly improved for devices with SubPc or SubNc as acceptor, compared to the conventional device (CuPc/C_60_). Two mechanisms, two-step exciton dissociation and Förster resonance energy transfer (FRET), can explain the contribution from the SubNc and SubPc acceptor layer to the photocurrent. Seriously, the FRET mechanism is comprised within two-step exciton dissociation from SubPc to SubNc, and the consequent charge transfer at the α-6T/SubNc interface. According to dipole-dipole interactions, FRET is a long-range exciton transfer process occurring between luminescent energy donor and absorptive energy acceptor [20]. From emissive donor molecules to acceptor molecules, the Förster transfer was investigated and given by Scully et al. [21]. It is found that the calculation results of Förster transfer are approximate when we assume the thickness of middle layer is 0 nm between bilayer and the three-layer device. That is to say, the distance between dissociated exciton and quenching layer is approximate with the Förster radius of energy transfer (R_0_) of SubPc or SubNc as an acceptor. Investigation of energy transfer in organic photovoltaic cells and impact on exciton diffusion length measurement was also researched [22,23,24] (pp. 2977–2979, pp. 5–6, pp. 765–768). The R_0_ about the value of 7.5 nm and 6.0 nm, depending on the spectral overlap between the emission of the energy donor and absorption of the energy acceptor, was extracted for exciton energy transfer from SubPc and SubNc, respectively. Finally, we also fabricated the three-layer structure to increase the photocurrent generation for combining three different materials, α-6T/SubNc/SubPc. The thickness of both acceptor layers was adjusted to gain the maximum PCE again. The three-layer cascade device, forming an important alternative to traditional OSCs, has a higher performance with J_SC_ of 9.88 mA/cm^2^, V_OC_ of 0.94 V and PCE of 4.35%. It is similar to the bilayer device which has low FF results from increased thickness of the SubNc layer. Moreover, an increase of photocurrent generation is observed in the three-layer device structure, in which the V_OC_ is similar to the bilayer device (α-6T/SubNc). Because of the approach of LUMO energy levels, the V_OC_ does not decrease suddenly. This results in an impressive consequence from the bilayer to three-layer device, exceeding the current technology of vacuum-evaporated single junction of organic photovoltaic device. Through a simple process of vacuum thermal evaporation, we can reduce the cost of manufacturing the solar cells. As a result, the device with SubNc has become the interlayer to replace fullerene. Similar to an energy-relay cascade structure with multiple donor materials, both SubNc and SubPc can be combined as acceptors in a three-layer device structure. The generated excitons are transferred from SubPc acceptor to the middle SubNc layer and consequently dissociated at the α-6T/SubNc interface. To optimize the efficiency of OSCs, the interaction between the active layer and anode should be considered. That is to say the SubNc layer simultaneously has two functions: including an energy acceptor for excitons generated in the SubPc layer and as a charge acceptor at the interface with α-6T.

## 4. Materials and Methods

All organic materials were obtained through commercial source and were used without further sublimation in this study. The materials in the device were alpha-sexithiophene (α-6T, 99.0%-Aldrich), boron subphthalocyanine chloride (SubPc, 99.0%-Lumintech), (CuPc, 99.0%-Aldrich), fullerene (C_60_, C_70_ 99.95%-Aldrich), and bathocuproine (BCP, 99.0%-Aldrich). The indium tin oxide-coated glass substrates (ITO-glass, with a sheet resistance of 7 Ω/sq) are sequentially cleaned through standard cleaning process of ultrasonic treatment in acetone, methanol, de-ionized (DI) water for 5 min each, and dried with nitrogen gas blow before deposition. The organic materials and Al (100 nm, as a cathode) were deposited by vacuum thermal evaporation below the pressure of 4.8 × 10^−6^ torr. The deposition rate of organic materials was at 0.03 ± 0.01 nm/s, and the cathode was deposited through a shadow mask, giving an active area of 6 mm^2^ at a deposition rate of 0.3 ± 0.03 nm/s. Deposition rate and film thickness were monitored by using quartz crystal oscillator. The current density-voltage (J-V) characteristics were measured with a power source meter (Keithley 2400, Keithley, Cleveland, AL, USA) under an illumination of 100 mW/cm^2^ with an AM1.5G sun simulator (Oriel 96000 150W Xe lamp, Newport, Taipei, Taiwan). The light intensity was calibrated by using a reference solar cell and meter (Oriel 91150, Newport, Taipei, Taiwan), and all measurements were carried out in air with encapsulation.

Investigations of microstructure and sheet resistance (R_s_) were carried out using X-ray diffraction (XRD, Rigaku/Ultima, Tokyo, Japan) and four point sheet resistivity (SRM103, Solar Energy Tech Ins, Suzhou, China), respectively. The work function of ITO was measured by using ultraviolet photoelectron spectroscopy (UPS, Sigma Probe, Thermo VG-Scientific, Waltham, MA, USA) with monochromatized He (21.2 eV) discharge lamp. The spectrum was taken for −5 V sample bias to separate the sample and the secondary edge for the analyzer. Devices were further measured by atomic force microscope (AFM, XE-70, Park Systems, Suwon, Korea) to analyze the surface morphology with 0.5 Hz scan rate probing an area of 20 × 20 μm^2^, which effectively reducing the speed at which the tip moves and allowing the system more time to adjust to sudden changes in height, and by spectrophotometer (UV-3900, Hitachi, Tokyo, Japan) to gauge the absorption spectrum of device. All measurements were carried out in air with encapsulation.

## 5. Conclusions

We have demonstrated that the simple three-layer architecture plays an important role in enhancing the PCE of OSCs by using various small molecule organic materials. The thin-film optical absorption spectrum is broadened theoretically for the entire spectrum of solar energy when the device comprising two non-fullerene acceptors and a donor was accomplished. We also accomplished the bilayer OSCs to understand the device performance with and without fullerene materials as acceptor. Both the surface roughness and work function are improved by inserting MoO_3_ anode buffer layer between the ITO and donor, at the same time, to explain the improved PCE and strengthened ability of the charge transportation. The V_OC_ of devices based on α-6T, SubNc, SubPc and CuPc as donor can be largely advanced by depositing MoO_3_ layer on ITO substrate, which results from less carrier recombination in the active layer and the improvement of work function in the anode. According to the two-step exciton-dissociating mechanism, both SubPc and SubNc can be combined as acceptors in the three-layer device structure by utilizing long-range FRET. The generated excitons are transferred from SubPc acceptor to the middle SubNc layer and consequently dissociated at the α-6T/SubNc interface. To optimize the efficiency of OSCs, the interaction between the active layer and anode should be considered. That is to say the SubNc layer simultaneously has two functions, including an energy acceptor for excitons generated in the SubPc layer and as a charge acceptor at the interface with a-6T.

## Figures and Tables

**Table 1:** The photovoltaic performance of bilayer organic solar cells (OSCs) (Donor/C_60_) is sorted and estimated the properties of devices with structure: ITO/MoO_3_ (10 nm)/Donor/C60 (30 nm)/Bathocuproine (BCP) (10 nm)/Ag (100 nm).

Material Thickness (nm)	J_SC_ (^mA^/_cm_^2^)	V_OC_ (V)	FF (%)	η (%)	R_S_ (Ω)	R_Sh_ (kΩ)
SubPc	4.57 ± 0.02	1.00 ± 0.01	55.27 ± 1.22	2.52 ± 0.10	31.71	1.125
9						
CuPc	3.41 ± 0.05	0.39 ± 0.02	54.00 ± 1.31	0.70 ± 0.08	23.63	0.96
20						
α-6T	3.67 ± 0.08	0.35 ± 0.02	55.30 ± 1.17	0.71 ± 0.05	23.53	0.96
40						

**Table 2:** The photovoltaic performance of bilayer OSCs (α-6T/Acceptor) is sorted and estimated the properties of devices with MoO_3_ (10 nm), BCP (10 nm), and Ag (100 nm).

Material Thickness (nm)	J_SC_ (^mA^/_cm_^2^)	V_OC_ (V)	FF (%)	η (%)	R_S_ (Ω)	R_Sh_ (kΩ)
α-6T	ITO/MoO_3_/α-6T (X nm)/C_70_ (30 nm)/BCP/Ag					
40	6.01 ± 0.07	0.36 ± 0.02	56.56 ± 1.02	1.23 ± 0.10	12.55	0.386
50	5.98 ± 0.02	0.38 ± 0.02	56.37 ± 1.04	1.28 ± 0.09	12.79	0.49
60	5.92 ± 0.05	0.39 ± 0.01	53.57 ± 1.42	1.24 ± 0.07	15.46	0.501
C_70_	ITO/MoO_3_/α-6T (40 nm)/C_70_ (X nm)/BCP/Ag					
20	6.35 ± 0.05	0.34 ± 0.01	58.30 ± 0.02	1.26 ± 0.04	9.77	0.563
25	7.05 ± 0.05	0.35 ± 0.02	58.28 ± 0.03	1.44 ± 0.09	9.47	0.454
30	6.26 ± 0.02	0.35 ± 0.02	57.66 ± 0.07	1.26 ± 0.08	11.23	0.517
32	6.03 ± 0.07	0.33 ± 0.01	57.01 ± 0.11	1.14 ± 0.04	10.31	0.416
SubNc	ITO/MoO_3_/SubNc (X nm)/C_70_ (30 nm)/BCP/Ag					
10	7.15 ± 0.13	0.68 ± 0.02	44.12 ± 1.85	2.15 ± 0.19	24.95	0.311
40	6.73 ± 0.11	0.72 ± 0.01	29.71 ± 1.77	1.44 ± 0.13	82.81	0.178
50	4.58 ± 0.18	0.70 ± 0.02	25.11 ± 1.82	0.81 ± 0.11	123.32	0.184

**Table 3:** The photovoltaic performance of bilayer OSCs (α-6T/Acceptor) is sorted and estimated the properties of devices with MoO_3_ (10 nm), BCP (10 nm), and Ag (100 nm).

Material Thickness (nm)	J_SC_ (^mA^/_cm_^2^)	V_OC_ (V)	FF (%)	η (%)	R_S_ (Ω)	R_Sh_ (kΩ)
SubPc	ITO/MoO_3_/α-6T (50 nm)/SubPc (X nm)/BCP/Ag					
9	6.01 ± 0.03	0.36 ± 0.03	56.56 ± 0.04	1.23 ± 0.10	12.55	0.386
5	5.98 ± 0.02	0.38 ± 0.01	56.37 ± 0.07	1.28 ± 0.04	12.79	0.49
18	5.92 ± 0.06	0.39 ± 0.02	53.57 ± 1.01	1.24 ± 0.10	15.46	0.501
α-6T	ITO/MoO_3_/α-6T (X nm)/SubPc (18 nm)/BCP/Ag					
50	4.14 ± 0.07	0.87 ± 0.03	50.70 ± 1.72	1.83 ± 0.13	53.64	1.45
60	3.83 ± 0.07	1.04 ± 0.03	64.14 ± 1.98	2.56 ± 0.21	17.24	2.689
SubNc	ITO/MoO_3_/α-6T (60 nm)/SubNc (X nm)/BCP/Ag					
12	5.75 ± 0.34	0.73 ± 0.04	51.66 ± 1.42	2.17 ± 0.32	64.86	0.608
16	5.68 ± 0.26	0.61 ± 0.05	51.79 ± 1.58	1.80 ± 0.29	12.46	0.481

**Table 4:** The photovoltaic performance of three-layer OSCs (α-6T/SubNc/SubPc) is sorted and estimated the properties of devices with MoO_3_ (10 nm), SubPc (18 nm), BCP (10 nm), and Ag (100 nm).

SubNc Thickness (nm)	J_SC_ (^mA^/_cm_^2^)	V_OC_ (V)	FF (%)	η (%)	R_S_ (Ω)	R_Sh_ (kΩ)
0	3.83 ± 0.05	1.04 ± 0.02	64.14 ± 1.55	2.56 ± 0.14	17.24	2.689
3	7.19 ± 0.02	0.96 ± 0.02	43.61 ± 1.24	3.01 ± 0.16	25.94	0.878
6	8.28 ± 0.01	0.93 ± 0.03	45.20 ± 1.31	3.48 ± 0.12	19.29	0.576
12	10.12 ± 0.06	0.94 ± 0.01	44.12 ± 1.48	4.20 ± 0.19	17.41	0.449
15	9.88 ± 0.01	0.94 ± 0.02	46.81 ± 1.17	4.35 ± 0.20	12.95	0.443
18	6.14 ± 0.03	0.93 ± 0.02	48.65 ± 1.06	2.78 ± 0.13	20.48	0.615
